# Resveratrol supplementation confers neuroprotection in cortical brain tissue of nonhuman primates fed a high-fat/sucrose diet

**DOI:** 10.18632/aging.100942

**Published:** 2016-04-09

**Authors:** Michel Bernier, Devin Wahl, Ahmed Ali, Joanne Allard, Shakeela Faulkner, Artur Wnorowski, Mitesh Sanghvi, Ruin Moaddel, Irene Alfaras, Julie A. Mattison, Stefano Tarantini, Zsuzsanna Tucsek, Zoltan Ungvari, Anna Csiszar, Kevin J. Pearson, Rafael de Cabo

**Affiliations:** ^1^ Translational Gerontology Branch, National Institute on Aging, NIH, Baltimore, MD 21224, USA; ^2^ Department of Physiology and Biophysics, Howard University, College of Medicine, Washington, DC 20059, USA; ^3^ Department of Biopharmacy, Medical University of Lublin, 20-093 Lublin, Poland; ^4^ Laboratory of Clinical Investigation, National Institute on Aging, NIH, Baltimore, MD 21224, USA; ^5^ University of Oklahoma Health Science Center, Oklahoma City, OK 73104, USA; ^6^ Graduate Center for Nutritional Sciences, University of Kentucky, Lexington, KY 40536, USA

**Keywords:** Rhesus monkeys, cDNA microarray, inflammation, endothelial nitric oxide synthase, brain vasculature

## Abstract

Previous studies have shown positive effects of long-term resveratrol (RSV) supplementation in preventing pancreatic beta cell dysfunction, arterial stiffening and metabolic decline induced by high-fat/high-sugar (HFS) diet in nonhuman primates. Here, the analysis was extended to examine whether RSV may reduce dietary stress toxicity in the cerebral cortex of the same cohort of treated animals. Middle-aged male rhesus monkeys were fed for 2 years with HFS alone or combined with RSV, after which whole-genome microarray analysis of cerebral cortex tissue was carried out along with ELISA, immunofluorescence, and biochemical analyses to examine markers of vascular health and inflammation in the cerebral cortices. A number of genes and pathways that were differentially modulated in these dietary interventions indicated an exacerbation of neuroinflammation (e.g., oxidative stress markers, apoptosis, NF-κB activation) in HFS-fed animals and protection by RSV treatment. The decreased expression of mitochondrial aldehyde dehydrogenase 2, dysregulation in endothelial nitric oxide synthase, and reduced capillary density induced by HFS stress were rescued by RSV supplementation. Our results suggest that long-term RSV treatment confers neuroprotection against cerebral vascular dysfunction during nutrient stress.

## INTRODUCTION

Worldwide incidence of obesity is at an all-time high, with more than 1.1 billion adults being classified as overweight or obese. The rise in obesity rates has a growing impact on the incidence of metabolic syndrome, cardiovascular disease, type-2 diabetes and cancer, and the cost of caring for obesity-related pathologies remains a huge economic burden, with $147-210 billion going towards the care of patients each year [1]. Epidemiological studies have shown a consistent link between increased body mass index at mid to late life and a stronger risk of late-onset dementia [2]. High-fat diet has been associated with central inflammation, brain insulin resistance, and cognitive decline leading to progressive neuro-degeneration [3], and resveratrol (RSV; 3, 4′, 5-trihydroxy-*trans*-stilbene) supplementation can attenuate central inflammation and improve memory deficits in HFD-fed mice [4].

RSV is a naturally occurring polyphenol compound that is found in a variety of foods including, but not limited to, wines, grape juice, nuts, and berries. Since 1940, RSV has generated considerable interest in the scientific community due to its ability to protect against a variety of diseases, including type-2 diabetes, cardiovascular disease, and cancers [5]. In addition, RSV has been recently proposed to play a critical role in the reduction of neuroinflammation caused by cytokines [6] and preservation of the cerebromicrovasculature density in cerebral endothelial cells [7, 8]. Obesity has been associated with marked increase in 4-hydroxynonenal (4-HNE), a membrane lipid peroxidation byproduct that induces mitochondrial oxidative stress leading to neuroinflammation and progressive neurodegeneration [9, 10]. RSV confers antioxidant protection by reducing formation of reactive oxygen species and counteracting the cytotoxic effects of 4-HNE in PC12 cells *in vitro* [11]. Additionally, RSV improves endothelial function and cerebral vascular density [12, 13] by increasing levels of vascular endothelial growth factor (VEGF) [14], which enhances angiogenesis [15] and attenuates ischemic brain damage [16].

Some clinical trials have been conducted to date to assess the role of RSV supplementation in brain function and/or neuroinflammation [17-20]. RSV dietary supplementation lowers infection-related neuroinflammation and impairment in working memory in mice [21] while it significantly increases cerebral blood flow and improves memory function in patients with an off-balance metabolism (reviewed in ref. [5]). More recently, it has been suggested that polyphenols are important to consume in old age as they may offer protection against neuroinflammation and help protect against cognitive decline [22]. The prefrontal cortex is the critical brain site for decision-making and other essential components of executive function [23] and middle-aged individuals with childhood-onset type 1 diabetes display poorer cognitive function [24]. Moreover, peripheral glucose dysregulation that commonly occurs with obesity is also associated with high rates of cognitive impairment [25-29]. Because of the inherent limitation of performing biochemical and histochemical analyses on post-mortem brain tissue from patients, the objective of this study was to set out a research protocol to compare various parameters of cerebral neuroinflammation in middle-aged male rhesus monkeys fed a high-fat, high-sucrose (HFS) diet without and with RSV supplementation for 2 years. Using this experimental model, we recently reported that RSV prevents pancreatic β-cells dedifferentiation [30], promotes metabolic and inflammatory adaptations in visceral white adipose tissue [31], and reduces the HFS-induced inflammation and stiffening of the central arterial wall [32]. In the present study, the prefrontal cortices on the same cohort of treated animals were used to test the hypothesis that RSV supplementation may prevent impairment in cerebral vasculature and confer neuroprotection through inhibition of oxidative and inflammatory pathways.

## RESULTS

### Assessment of resveratrol accumulation in monkey cerebrospinal fluid

**Figure 1:**
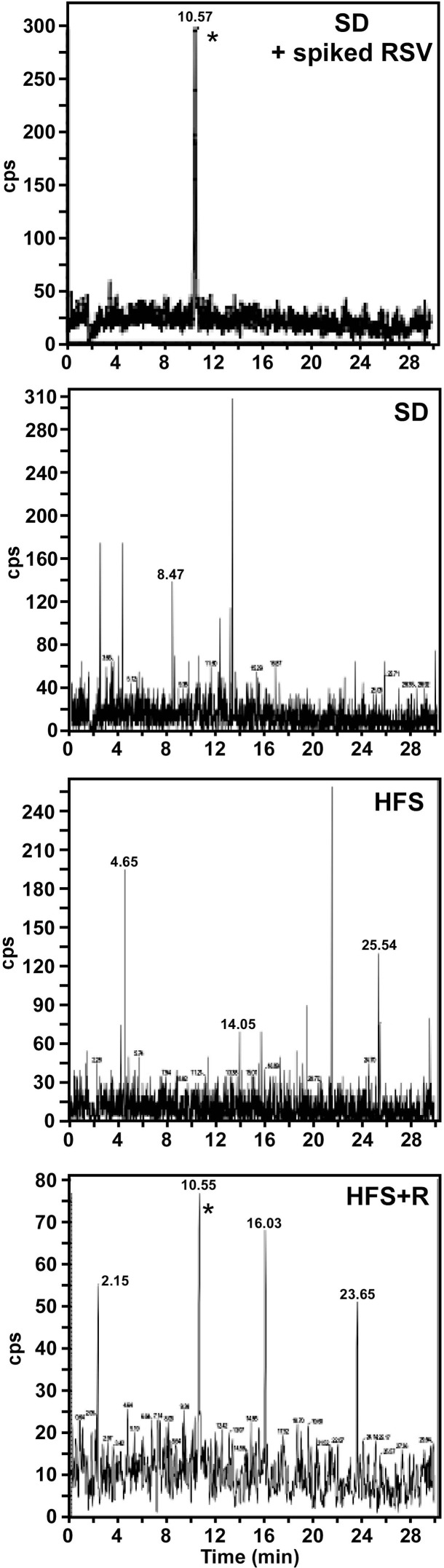
Accumulation of RSV in monkey CSF At the time of sacrifice, CSF from SD, HFS and HFS+R animals was collected and the amount of unconjugated RSV measured by MS/MS analysis. Upper panel, a CSF sample from SD control was spiked with 2.5 ng/ml RSV. The detection of RSV is indicated by *.

Adult male rhesus monkeys (average age at baseline 10.5 ± 0.4 years) were fed a SD, HFS or HFS supplemented with RSV (HFS+R) diet for 24 months. Following euthanasia, RSV was detected in CSF of rhesus monkey on HFS+R diet, but not in SD- or HFS-fed animals (Fig. 1). No attempts were made to identify metabolites of RSV; however, we previously reported the detection of RSV and resveratrol-3-*O*-sulfate in the serum of HFS+R-fed animals, but not of resveratrol-4′-*O*-glucuronide nor 3-*O*-glucuronide, which were below the minimum quantitation limits [30]. There are no data available about concentrations of RSV in CSF in year 1.

### Resveratrol supplementation alters gene expression profiles in cerebral cortex of HFS-fed monkeys

**Figure 2:**
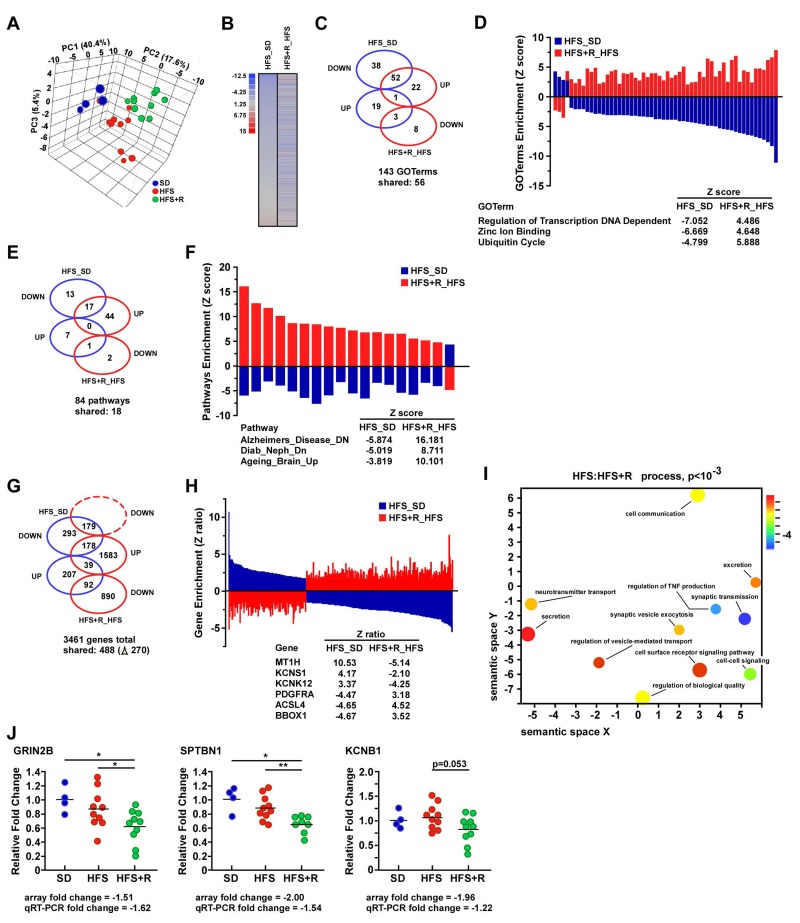
Resveratrol supplementation elicits differential gene expression profiles in the frontal cerebral cortex of HFS-fed rhesus monkeys (**A**) Visualization of the principal component analysis (PCA) performed on gene expression data sets from the brain of rhesus monkeys maintained on standard diet (SD), high-fat, high-sugar diet (HFS) and HFS supplemented with resveratrol for 2 years (HFS+R). (**B**) Heat maps representing gene expression profile comparing genes significantly up (red) and downregulated (blue) in HFS vs. SD-fed controls and HFS+R vs. HFS. (**C**) Venn diagram illustrating the number of significantly up and down regulated GO terms observed in neocortex from HFS vs. SD (blue symbols) and HFS+R vs. HFS-fed monkeys (red symbols). (**D**) Enrichment of the 56 shared GO terms visualized using a two-dimensional graphical representation of HFS vs. SD (blue bars) and HFS+R vs. HFS (red bars). A list of three shared GOTerms differentially expressed between the two pairwise comparisons is provided. (**E**) Venn diagram illustrating the number of significantly up and down regulated pathways between the two pairwise comparisons. (**F**) Enrichment of the 18 shared pathways visualized using a two-dimensional graphical representation of HFS vs. SD (blue bars) and HFS+R vs. HFS (red bars). A list of three shared pathways differentially expressed between the two pairwise comparisons is provided. (**G**) Venn diagram illustrating the number of significantly up and down regulated genes between the two pairwise comparisons. (**H**) Enrichment of the 270 shared genes visualized using a two-dimensional graphical representation of HFS vs. SD (blue bars) and HFS+R vs. HFS (red bars). A list of six shared genes differentially expressed between the two pairwise comparisons is provided. (**I**) 5170 genes from the filtered brain dataset (HFS:HFS+R comparison) were ranked according to their differential expression and given as input to GOrilla. The resulting enriched GO terms were visualized using a two-dimensional graphical representation with color coding reflecting their degree of enrichment/depletion (blue being the strongest). Additional comparisons (HFS vs. SD) on the same filtered dataset highlight the enrichment of relevant GO terms, such as “Synapse part”, “Axon part”, “Oxidation-reduction process” and “Response to axon injury”. (**J**) mRNA expression analysis in brain cortex by quantitative RT-PCR. Relative expression values were normalized to those of SD-fed control monkeys and represented as scatter plots. Although the fold changes in *GRIN2B*, *SPTBN1* and *KCNB1* expression were small [less than a 2-fold change], these were in good agreement with the quantitative RT-PCR data. SD (n=4); HFS (n=10); HFS+R (n=10). *, **, p <0.05 and 0.01, respectively.

Whole genome microarray analysis was performed on cerebral cortex tissues of middle-aged male rhesus monkeys fed for two years either with SD, HFS or HFS+R diet. Principal component analysis (PCA) demonstrated a clear separation of the three experimental groups (Fig. 2A). The dissimilarity in the pattern of gene expression between the HFS_SD and HFS+R_HFS pairwise comparisons is graphically represented as heat maps (Fig. 2B), and indicates that RSV supplementation provides a transcriptional profile distinct from that elicited by HFS diet alone. Gene ontology analyses of biological processes highlighted 56 out of a total of 143 GO terms that were shared between HFS_SD and HFS+R_HFS pairwise comparisons (Fig. 2C). Of these, there were 55 GO terms (55/56) enriched by genes that were differentially expressed between HFS and HFS+R diets, with “Regulation of transcription DNA dependent”, “Zinc ion binding” and “Ubiquitin cycle” among the top expressed GO terms (Fig. 2D). Significantly regulated genes were then organized into functional pathways using parametric analysis of gene-set enrichment (PAGE) analysis. Of the 84 genesets modified between the two pairwise comparisons, 18 were shared and populated by genes that were differentially expressed by HFS and HFS+R (Fig. 2E). “Alzheimers_Disease_ Down”, “Diab_Neph_Down” and “Ageing_Brain_Up” were among the pathways that were significantly downregulated by HFS and markedly reversed with RSV supplementation (Fig. 2F). Among the 3461 genes whose expression was significantly modified under these experimental conditions, 14.1% of the transcripts (488/3461) were shared in response to HFS and HFS+R, of which 218 genes (corresponding to 44.7% of the shared transcripts) moved in the same direction by both interventions (39 and 179 genes up- and down-regulated, respectively), while 270 genes (55.3% of the shared transcripts) were reciprocally altered by HFS and HFS+R (Fig. 2G). Fig. 2H depicts graphically the enrichment of these reciprocally regulated genes associated with the HFS_SD and HFS+R_HFS pairwise comparisons. The gene encoding the metallothionein MT1H transcript, which is inducible by ROS-mediated generation of lipid peroxides [33], was the most upregulated in response to HFS compared to SD, with a Z-ratio of +10.53 (Fig. 2H). Other genes upregulated by HFS included *KCNS1* and *KCNK12*, two voltage-gated potassium channel modulatory subunits present in the brain [34, 35]. This finding is consistent with recent evidence showing that inflammation can alter expression of neuronal potassium channel subunit mRNAs [36]. *BBOX1* was the most downregulated transcript in response to HFS, with a Z-ratio of −4.67, and represented one of many genes that exhibited a reciprocal pattern of expression with RSV treatment (Fig. 2H). The *BBOX1* gene encodes gamma butyrobetaine hydroxylase, an enzyme implicated in the L-carnitine biosynthetic pathway in the brain [37]. Interestingly, the maturation of *BBOX1* mRNA is nutritionally regulated to adjust L-carnitine biosynthesis to the energy supply [38]. The raw data file and the filtered, normalized results are available online in the Gene Expression Omnibus, accession number GSE70255.

Semantic indexing heatmap analysis [39, 40] of the monkey cortical brain microarray dataset identified enriched GO terms that were implicitly involved in inter-related functional networks associated with synaptic transmission, axon injury and oxidation-reduction processes, when comparing HFS to HFS+R cohorts (Fig. 2I). Several genes whose transcripts have been associated with cortical development and neuronal transmission were impacted by RSV supplementation in HFS-fed monkeys, and included *CHRNA5*, *GRIN2B* and *KCNB1* [41-43]. Expression of a vital component in actin-cytoskeleton organization in axon, *SPTBN1* [44], was also affected in the HFS+R cohort. Of significance, HFS diet caused an increase in the expression of *CST3*, a transcript that correlates positively with cognitive impairment in the elderly [45], which did not occur with RSV supplementation. The expression of *GRIN2B, SPTBN1* and *KCNB1* transcripts relevant to CNS was verified by quantitative RT-PCR analysis (Fig. 2J). The validated genes belong to the following two GO classes: “Synapse part (GO:0044456)” – *GRIN2B* (glutamate receptor, ionotropic, n-methyl d-aspartate 2b) and *KCNB1* (potassium voltage-gated channel, shab-related subfamily, member 1), and “Axon part (GO:0033267)” – *SPTBN1* (spectrin, beta, non-erythrocytic 1).

Expression of *GRIN2B* and *SPTBN1* mRNA levels was significantly lower in HFS+R vs. SD and HFS groups, while the reduction in *KCNB1* mRNA did not quite reach conventional levels of statistical significance (p= 0.053). Nevertheless, the changes in the mRNA transcript levels between the microarray and quantitative RT-PCR data were identical in terms of direction and magnitude of changes (Fig. 2J).

### Resveratrol supplementation improves regional brain capillary density in cerebral cortex of HFS-fed monkeys

**Figure 3:**
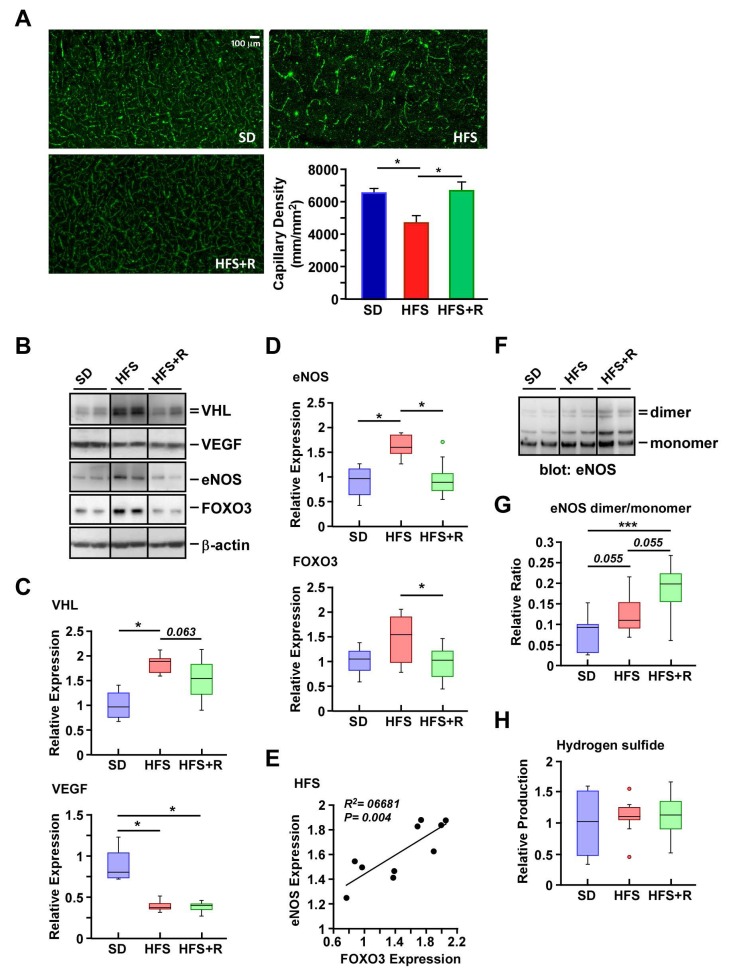
Resveratrol treatment improves capillary density in the cerebral cortex of HFS-fed rhesus monkeys (**A**) Representative images of cerebral cortical brain capillary staining, and their respective quantification. (**B**) Solubilized cerebral cortex extracts from SD-, HFS- and HFS+R-fed animals were resolved by SDS-PAGE under reducing conditions, electrotransferred onto nitrocellulose membranes and subjected to immunoblotting using the indicated primary antibodies. Representative signals associated with bands of interest are shown, including that of *b*-actin, which was used as loading control. All 24 brain samples were ran on a single gel (Fig. S1). Nitrocellulose membrane staining was also carried out with Ponceau S dye for protein detection. (**C**) Signals associated with VEGF and VHL proteins were normalized and represented as box plots. (**D**) Signals associated with eNOS and FOXO3 proteins were normalized and represented as box plots. (**E**) Scatterplot exploring the significant association between eNOS and FOXO3 protein expression with HFS feeding. (**F**) SDS-PAGE was performed at 4°C to enable the separation of eNOS dimers and monomers. Immunoblot analysis was performed using anti-eNOS antibody. (**G**) Ratios of dimeric and monomeric eNOS species were calculated and represented as box plots. (**H**) Measurement of H2S levels in monkey brain homogenates. SD (n=4); HFS (n=10); HFS+R (n=10). *, p < 0.05.

Microscopic visualization was performed to determine the effect of diets on cerebral vasculature in rhesus monkeys. A consistent pattern of distribution was observed in the vascularization of the neocortex of SD-fed animals and, by comparison, was substantially less in HFS brain (Fig. 3A). RSV supplementation offered significant protection against the HFS-mediated loss in capillary density in the neocortex. We then investigated the expression of a series of proteins implicated in angiogenesis, including von Hippel-Lindau protein (pVHL), VEGF and endothelial nitric oxide synthase (eNOS) using Western blot analysis. *VHL* encodes the ubiquitin ligase pVHL, and HFS diet caused a significant increase in pVHL levels with concomitant reduction in the amount of VEGF protein (Fig. 3, B and C), which could explain the lower vascularization in the neocortex of HFS-fed rhesus monkeys (Fig. 3A). A trend toward reduced accumulation of pVHL without upregulation of VEGF was observed in HFS+R compared to HFS cerebral cortex extracts (Fig. 3, B and C).

Cerebral blood flow requires the generation of nitric oxide and eNOS-derived nitric oxide appears to exhibit neuroprotection under physiological conditions (review, see Ref. [46]). Some stimuli trigger the production of superoxide instead of nitric oxide through increased monomerization of eNOS and alteration in its enzymatic function. This phenomenon, termed eNOS uncoupling, exacerbates oxidative stress and the depletion of nitric oxide, together with neuronal apoptosis and ROS release after brain injury [47]. We therefore examined whether the abundance and/or monomer-dimer equilibrium of eNOS would be impacted by dietary stress. There was a significant increase in the amount of eNOS protein with a trend toward higher expression of its transcriptional activator FOXO3 in response to HFS as compared to SD-fed controls (Fig. 3, B and D). A positive linear relationship between eNOS levels and FOXO3 expression was established in the HFS cohort (p < 0004; Fig. 3E), and this correlated well with a deficit in brain capillary density. Diet supplementation with RSV significantly reversed the HFS-mediated increase in the steady-state levels of FOXO3 and eNOS proteins (Fig. 3, B and D). Despite the fact that eNOS expression was actually increased by HFS, the eNOS dimer/monomer ratio was comparable to that of SD-fed controls while RSV treatment caused a significant elevation in the ratio (p<0.001, Fig. 3, F and G). Because the dimeric eNOS is the only form of the enzyme that produces nitric oxide [48], these results are consistent with improved cerebral blood flow in the presence of increased capillary density due to RSV supplementation.

The endogenous level of hydrogen sulfide (H2S) dilates cerebral vessels by activating smooth muscle cell plasma membrane ATP-sensitive K^+^ channels. It also increases eNOS activity by promoting the formation of eNOS dimers [49]. Here, large intra-individual variations in H2S levels in SD, HFS and HFS+R groups were observed (Fig. 3H), making it impossible to determine whether the RSV-mediated protection in cerebral vasculature of HFS-fed rhesus monkeys stemmed from increases in H2S tissue concentration in the brain.

### Resveratrol supplementation and inflammatory state in the cerebral cortex of HFS-fed monkeys

**Figure 4:**
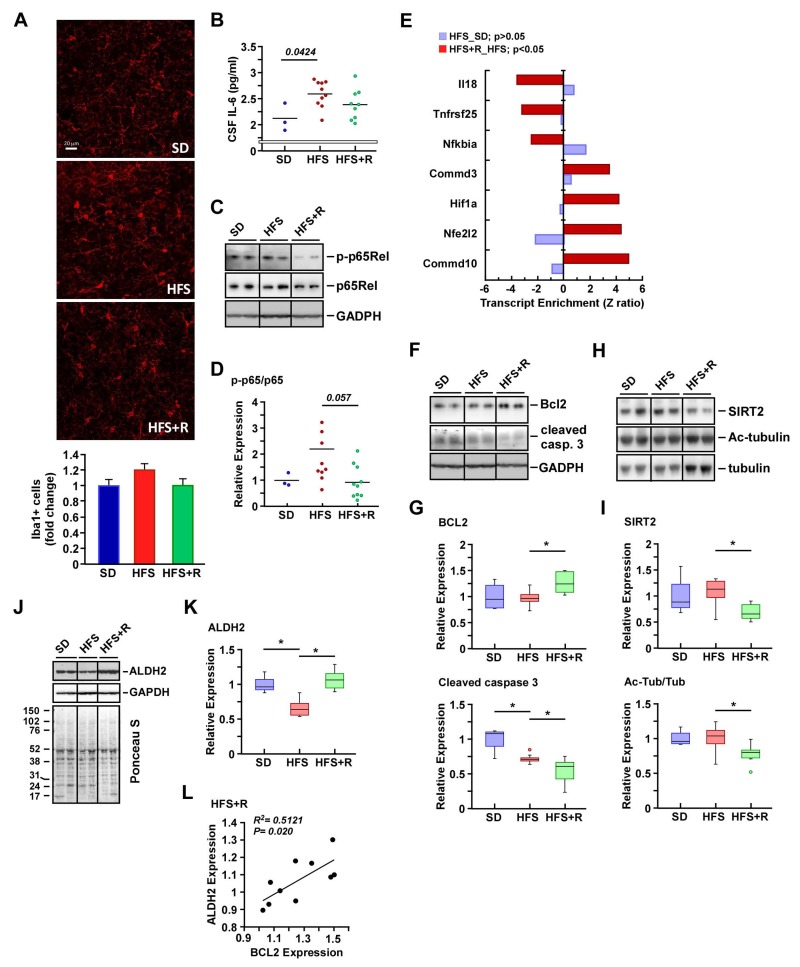
Effects of resveratrol on neuroinflammation in the cerebral cortex of HFS-fed rhesus monkeys (**A**) Representative images of Iba1-positive cells in cerebral cortical brain sections (original magnification: 40x), and their respective quantification. (**B**) IL-6 levels in CSF. (**C-K)** Solubilized cerebral cortex extracts from SD-, HFS- and HFS+R-fed animals were resolved by SDS-PAGE under reducing conditions, electrotransferred onto nitrocellulose membranes and subjected to immunoblotting. All 24 brain samples were ran on a single gel (Fig. S1). (**C**) Representative signals associated with phosphorylated and total forms of p65Rel and that of GAPDH, which was used as loading control. (**D**) The ratios of phosphorylated/total p65Rel proteins are represented as scatter plots. (**E**) Enrichment of a select group of transcripts implicated in NF-κB signaling both in HFS vs. SD and HFS+R vs. HFS pairwise comparisons. (**F**) Representative signals associated with BCL2 and cleaved caspase 3, and that of GAPDH, which was used as loading control. (**G**) Signals associated with BCL2 and cleaved caspase 3 proteins were normalized and represented as box plots. (**H**) Representative immunoblot analysis for SIRT2, acetylated tubulin, and total tubulin levels. (**I**) Signals associated with bands of interest were normalized to Ponceau S staining and represented as box plots. (**J**) Representative signals associated with ALDH2 and GAPDH, the latter being used as loading control. Membrane staining was also carried out with Ponceau S dye for protein detection. The migration of molecular-mass markers (values in kilodaltons) is shown on the left of the stained membrane. (**K**) Signal associated with ALDH2 was normalized to GAPDH and represented as box plots. (**L**) Scatterplot exploring the significant association between ALDH2 and BCL2 protein expression with HFS+R feeding. SD (n=4); HFS (n=10); HFS+R (n=10). *, p < 0.05.

There was no significant difference in the number of cells positive for the microglial marker Iba1^+^ among the groups (Fig. 4A). Despite the low sample size in the SD group, evidence was provided for higher levels of IL-6 in CSF after HFS feeding (p = 0.0424, Fig. 4B) and for the lack of difference between the HFS and HFS+R groups. Additionally, the increased ratio of phospho-active to total form of NF-κB p65Rel in response to HFS vs. SD was not conclusive (Fig. 4, C and D), and neither was the difference in the activation state of p65Rel between the HFS+R group and HFS (p = 0.057, Fig. 4, A and D).

A critical mechanism of transcriptional suppression of NF-κB involves the copper metabolism MURR1 domain containing (COMMD) proteins through promotion of the ubiquitination and degradation of NF-κB subunits [50]. Further analysis of the microarray dataset revealed a significant increase in COMMD10 and COMMD3 transcripts with a concomitant reduction of transcripts corresponding to known NF-κB targets, such as NFKBIA, TNFRSF25 and IL18, in the cerebral cortex of HFS+R versus HFS-fed animals (Fig. 4E). The BCL2 family of genes is transcriptionally regulated by NF-κB in the brain [51], and we demonstrated here that diet supplementation with RSV significantly increased the expression of the antiapoptotic protein BCL2 with marked reduction in the active (cleaved) form of caspase 3 as compared to HFS (Fig. 4, F and G). Low caspase 3 cleavage was also observed in the HFS group as compared to SD.

Various members of the sirtuin class of NAD(+)-dependent, protein deacetylase family have been found to be involved in processes related to the regulation of inflammation and neurobiology. Whilst SIRT1 has been by far the most extensively studied sirtuin, there has been recent interest in cytoplasmic SIRT2, particularly due to its abundance in the CNS and its expression increasing with age [52]. SIRT2 plays an important role for neurocognitive ability in adult, middle-aged mice and few studies have demonstrated that inhibition of SIRT2 reduces the aggregation of α-synuclein via modulation of tubulin activity to mitigate Parkinson's toxicity [53, 54]. Because high-fat diet is a known inducer of NF-κB-mediated neuroinflammation in cerebral cortex [55], we examined whether RSV contributes to the regulation of cerebral expression of SIRT2 and its activity as a bona fide α-tubulin deacetylase [56] in response to HFS. Immunoblotting revealed significant reduction in SIRT2 protein levels caused by RSV supplementation (Fig. 4H and I upper panel, Supplemental Fig. 1). The decrease in SIRT2 levels was accompanied by higher expression of α-tubulin, which, in turn, led to a significant reduction in the acetylated/total tubulin ratio in the HFS+R cohort compared to the HFS group (Fig. 4H and I lower panel, Supplemental Fig. 1).

Mitochondrial aldehyde dehydrogenase (ALDH2) is implicated in the metabolism and inactivation of reactive aldehydes, which represent major end products of lipid peroxidation and contributing factors in diet-induced neuronal oxidative stress. An association has been reported between brain ALDH2 levels and protection against ischemic stroke [57]. Moreover, mice lacking ALDH2 exhibit endothelial dysfunction, age-related cognitive impairment and Alzheimer's disease [58]. We therefore measured the abundance of ALDH2 protein in the cerebral cortex of HFS-fed rhesus monkeys and found a significant reduction in its expression level (Fig. 4J and K, Supplemental Fig. 1). RSV supplementation helped preserve the amount of ALDH2 despite HFS-mediated oxidative stress, which may indicate substantial protection against neocortex damage. Consistent with this, a positive linear relationship between ALDH2 levels and expression of the anti-apoptotic BCL2 protein was established in the HFS+R cohort (Fig. 4L). Taken together, the results indicate that RSV supplementation may overcome HFS-induced cerebrovascular dysfunction and neuroinflam-mation by preventing the destabilization of ALDH2.

## DISCUSSION

The ability of RSV supplementation to promote a shift away from diet-induced brain inflammation towards increase in neuronal health in a nonhuman primate model of obesity highlights important implications for future translational studies aimed at the prevention of neuronal damage and brain dysfunction. The fact that RSV can exert neuroprotective effect in various animal models under high-caloric conditions is already established; however, our observations in Rhesus monkeys represent an unique opportunity to validate these emerging concepts and for a period far longer than any clinical trials to date.

Our study on the long-term effect of RSV supplementation in metabolically impaired monkeys was initiated several years ago and has led to a number of significant observations including the decline in arterial wall stiffening and inflammation [32], lower pancreatic islet β-cell dedifferentiation [30] and improved insulin signaling in adipose tissue [31]. Here, we show that the CSF levels of IL-6 were significantly higher in HFS monkeys than in the SD group together with reduction in brain capillary density and amount of ALDH2 in the cerebral cortex. The gene profiling in the cerebral cortex of the HFS group resembled that previously described for physical trauma brain injury (TBI) in mice [59], with the most striking changes involving the regulation of functional pathways, notably “*StemCell Embryonic Up*”, “*StemCell Neural Up*”, “*Alzheimer's Disease Up*” and “*Alzheimer's Disease Dn*”. RSV was readily detected in CSF samples from the HFS+R group, along with the recovery of brain capillary density and protection against HFS-induced alteration in NF-κB transcriptional activity.

HFS is associated with alterations in cerebral vasculature, and consumption of RSV ameliorates endothelial hyperpermeability in HFS-fed rats partly via eNOS regulation [60]. We showed that the dysfunction of eNOS through homodimeric uncoupling provided a mechanism for diet-induced brain insult. Importantly, eNOS dimer stabilization was observed in the cerebral cortex of the HFS+R group vs HFS, and RSV supplementation was found to attenuate HFS-mediated induction of eNOS protein and its transcriptional activator FOXO3. The higher levels of eNOS in the HFS group may be an attempt in trying to improve blood flow in the presence of low capillary density due to HFS. Notable are the studies in which H2S was reported to affect eNOS function while playing a cardinal role in the protection of neurons from secondary injury by functioning as an anti-oxidant and anti-inflammatory mediator (reviewed in Ref. [61]). We could not demonstrate the contribution of H2S in modulating eNOS dimer/monomer ratio and/or activity due to large intra-individual variations among the groups. Similar to the protective effects of RSV in the monkey central arterial wall [32], RSV may potentially reduce the HFS-induced cerebrovascular inflammation by limiting transport of circulating cytokines across the blood-brain barrier, lending credence to the report of Chang et al. [62], who showed that high-fat diet supplementation with RSV for 8 weeks significantly reduced the disruption of the blood-brain barrier while protecting brain neurons in the mice from apoptotic insults. Based upon these results, it would have been interesting to perform a long-term dietary stress first and then initiate RSV supplementation in order to restore the cortical brain capillary density and protect against neuronal brain insults.

Administration of RSV has been found to greatly attenuate global cerebral ischemia by reducing levels of pro-inflammatory cytokines and promoting a reduction in associated plaque formation in the rat brain [63]. In addition, the ability of RSV to acutely inhibit microglial activation is consistent with its anti-inflammatory activity against various neurological disorders in experimental mouse models [64] and in a mouse model of physical TBI [65]. Here, RSV consumption had only minimal impact on both the activation of macrophage/microglial cell population and the steady state levels of active NF-κB p65 subunit when compared to the HFS group. In contrast, there was significant difference in the expression of genes involved in the inflammatory process between the HFS and HFS+R groups, with the RSV-treated group exhibiting anti-inflammatory protection. The deacetylase SIRT1 is a putative target of RSV action, and significant increase in *Commd* expression has been reported in the muscle of mice treated with the small SIRT1 activator, SRT2104, which was accompanied by SIRT1-mediated inhibition in NF-κB transcriptional activity [66]. Our results indicated that the SIRT1 content in the monkey cerebral cortex was comparable among the three experimental groups (data not shown), although acetylation levels of protein targets of SIRT1 may have been impacted. Nevertheless, we focused our attention toward SIRT2. SIRT2 functions as the predominant microtubule deacetylase in mature mouse cortical neurons, and impairment in tubulin acetylation/deacetylation activity has been associated with neuropathological features [67, 68]. The localization of SIRT2 along the microtubule network and the previous observation that regulation of neuronal morphology depends on the ability of microtubules to mediate intracellular transport and control local signaling events led us to the discovery of a significant reduction in SIRT2 protein expression in response to RSV supplementation. Unexpectedly, a decrease in the acetylated/total α-tubulin ratio through induction in α-tubulin levels was observed, which suggested to us that other tubulin deacetylases such as HDAC6 [69] were refractory to RSV treatment. SIRT1 protein contains no significant tubulin deacetylase activity (see Fig. 3C in [56]). Limited studies in mice using genetic and pharmacological approaches to disrupt SIRT2 expression and/or activity have generated divergent outcome [70, 71]. Genetic knockout of SIRT2 in a Huntington's disease mouse model showed no improvement in tubulin acetylation and the progression of neurological pathologies [70], whereas treatment with a SIRT2 inhibitor for up to 14 weeks confers neuroprotection against Huntington's related toxicity [71]. In our study, the benefits of treating HFS-fed monkeys with RSV for 2 years have been achieved through partial depletion of SIRT2 protein levels and improved neuroinflammation even though the identity of the molecular targets of RSV remain unknown.

The microarray experiment has identified a number of genes that were impacted by RSV supplementation such as voltage-gated potassium channel modulatory subunits, the glutamate receptor and axonal spectrin. Additionally, gene sets related to oxidative stress and activation of NF-κB target genes were found to be downregulated in the HFS+R group as compared to HFS. It is interesting that chronic *in vivo* NFAT-dependent transcription leads to pathological formation of neurofibrillary tangles in a mouse model of Alzheimer's disease, which can be attenuated by polyphenols [**72**]. Moreover, RSV supplementation prevents pathological cardiac hypertrophy by inhibiting the transcription factor NFAT [73]. It remains to be established whether NFAT activation occurs in cerebral cortex of HFS-fed monkeys and returns to basal steady state levels following diet supplementation with RSV.

Protection against progressive apoptotic cell death is a desirable feature when considering the impact of long-term dietary stress. The increase in inflammation and vascular dysfunction that results from obesity and metabolic disorders led us to examine the expression of apoptosis markers in monkey cerebral cortex. As expected, a clear effect on BCL2 levels and amount of active caspase 3 was observed in the HFS+R group as compared to HFS, thus providing evidence of the anti-apoptotic role of RSV against HFS-induced apoptosis. Though there was less active caspase 3 in cerebral cortex of the HFS group as compared to SD, BCL2 was expressed at similar levels between the two groups, indicating that the 2-year regimen with HFS may not have been sufficient to promote overt neuro-inflammation and apoptosis-related events. Alternatively, the indicated difference in active caspase 3 levels between the SD and HFS groups may not be conclusive due to low SD sample size. ALDH2 expression was significantly reduced by HFS, but normalized with RSV supplementation in parallel with the increase in BCL2 levels, and this can explain the compensatory changes to defend the brain against diet-induced oxidative damage leading to neuronal apoptosis.

When combined with the observation that a 6-month diet supplementation with RSV improves memory in overweight older individuals [20], it appears likely that attenuation of diet-induced neuroinflammation by brain resident cells, e.g., activated microglia and astrocytes, and perivascular macrophages may provide some of the cognitive benefits of RSV. A 52-week administration of 1000 mg RSV twice daily in individuals with Alzheimer's disease has been found to be well tolerated and resulted in changes in the trajectory of some biomarkers compared to placebo [74].

Altogether the present work outlines the preclinical efficacy of RSV supplementation in the prevention or delay of cerebral vascular dysfunction induced by dietary stress in rhesus monkeys, a highly relevant model of human health and disease. These findings add to an expending literature investigating the benefits of RSV in combatting various ailments [5].

## METHODS

### Animals and diets

Twenty-four adult (7-13 year old) male rhesus monkeys (*Macaca mulatta*) were housed at the NIH Animal Center. Animals were housed individually in standard nonhuman primate caging, in a temperature-regulated room (25 ± 1°C), humidity at 60 ± 20%, on a 12h light/12h dark cycle (lights on from 6:00 to 18:00) with *ad libitum* access to drinking water. All monkeys had extensive visual, auditory, and olfactory but limited tactile contact with monkeys housed in the same room. Monkeys were monitored minimally 3 times daily by trained animal-care staff. All procedures and animal care were conducted in accordance with the National Institutes of Health (NIH) Guide for the Care and Use of Laboratory Animals in an AALAC-accredited facility.

During baseline assessment, all monkeys were maintained on standard NIH monkey chow (Purina Mills). After baseline assessment, monkeys were quasi-randomized to one of three dietary regimens such that average age and bodyweight did not differ significantly between groups: control diet group fed a healthy standard diet (SD) with 13% kcal in fat and less than 5% sucrose by weight, high fat and sugar diet (HFS) group fed with 42% kcal in fat and 27% sucrose by weight (Harlan, Teklad, Indianapolis, IN, USA), and HFS + RSV (HFS+R) group fed with the same HFS diet plus a flavored primate treat (BioServ, Flemington, NJ, USA) containing 40 mg RSV (DSM Nutritional Products, Parsippany, NJ, USA) twice daily for one year. For the second year of the study, the RSV dose was increased to 240 mg twice a day to raise plasma RSV levels at 27.7 ± 8.6 ng/ml (29). No adverse effects to the RSV were evident. Monkeys in non-RSV groups received placebo treats. The RSV dose (240 mg) was derived from the protective dose reported in mice (22 mg/kg) [75] and adjusted by allometric scaling to an average monkey body weight of 12.1 kg.

All groups received 2 meals per day in allotments that represent *ad libitum* feeding. The HFS diet was formulated to cause weight gain and symptoms of metabolic syndrome, such as increased abdominal circumference, higher serum insulin levels after an intravenous glucose challenge, higher LDL-cholesterol but lower HDL-cholesterol levels compared to baseline levels, as well as lower insulin sensitivity after 24 months on a HFS diet [30, 31].

### Tissue sample collection, brain sectioning and immunofluorescence

At the end of the two-year dietary treatment period, monkeys were anesthetized with ketamine (7-10 mg/kg, IM). Following collection of blood and cerebrospinal fluid (CSF), animals were euthanized by an overdose of pentobarbital (50 mg/kg) and perfused with cold lactated Ringer's solution. Brain and various other organs and tissues were harvested immediately and either frozen at −80°C or fixed for further analyses. All experimental procedures were approved by the Animal Care and Use Committee of the NIA Intramural Program. The final protocol number for this project was #379-LEG-2010.

Brains were cut into 8 × 1-cm thick coronal blocks using a brain matrix. Each block was then cut sagittally along the midline. The entire left side of the brain was placed in 4% paraformaldehyde. Twenty-four to forty-eight hours after placement in paraformaldehyde, left hemispheres were cryoprotected in 20% sucrose solution for one week, then frozen in Cryo-Gel (Electron Microscopy Sciences, Hatfield, PA, USA) for sectioning. Coronal sections of 70 μm were cut through the cortex blocks and stored free-floating in cryopreservative solution (25% glycerol, 25% ethylene glycol, 25% 0.2 M phosphate buffer, 25% water) at −20°C. After washing, sections were treated with Proteinase K solution for antigen retrieval and were then treated with 1% of sodium-borohydride solution. Brain sections were blocked in 5% BSA/TBS (supplemented with 0.5% Triton X-100, 0.3 M glycine and 1% fish gelatin) and then immunostained with mouse anti-CD31 (Abcam, Cambridge, MA, USA) and rabbit anti-Iba1 (Wako, Richmond, VA, USA) antibodies at 4°C. After extensive washing, brain sections were labeled with donkey anti-mouse IgG, Alexa Fluor 488 and donkey anti-rabbit IgG, Alexa Fluor 568 both from Life Technologies (Grand Island, NY, USA), and then counterstained with 4′,6-diamidino-2-phenylindole. The mounted slides were imaged by both fluorescence microscopy and on a Leica confocal microscope using 20x and 40x objectives.

Capillary density in the cortex was quantified as the length of blood vessels <10 μm in diameter per volume of tissue using ImageJ software (NIH, Bethesda, MD, USA). The total length of capillaries was divided by the cortex area to obtain capillary density (length per area of tissue). Immunofluorescence labeling for Iba1 was used to identify microglia in the brain. The relative number of Iba1-positive microglia per region of interest in the cortex was calculated. The areas were analyzed using the area measurement tool of the MetaMorph software (version 7.7.9.0). In each animal, four randomly selected fields from the cortex were analyzed in six nonadjacent sections for both staining. The experimenter was blinded to the groups and animal treatments throughout the analysis.

### RNA extraction, cDNA synthesis, and quantitative PCR

Total RNA was extracted from frozen monkey temporal neocortex and purified using RNeasy Mini Kit (Qiagen, Valencia, CA, USA). A total of 0.5-1 μg total RNA was converted into first-strand cDNA using the High-Capacity cDNA reverse transcription kit with random hexamers, according to the manufacturer's instructions (Applied Biosystems, Foster City, CA, USA). SYBR Green PCR Master Mix (Applied Biosystems) was used for quantitative PCR reactions, and samples were analyzed on the 7500 Real-Time PCR System (Applied Biosystems). Oligonucleotide sequences were as follows:

GRIN2B (XM_001088140.2), 5′-CCCAGATCCT CGATTTCATT-3′ and 5′- GCCAAACTGGAAGAA CATGG-3′;
SPTBN1 (NM_001266968.1), 5′- CCTCTGATCGCA AAGCCAAGAC-3′ and 5′- CCACTCGTGTTTCCGA TTGAG-3′;
KCNB1 (NM_001265653.1), 5′-ACCGAATCCAACA AGAGCGT-3′ and 5′-TGCAAGCTTAAGGAT GCGGA-3′;
glyceraldehyde phosphate dehydrogenase (GAPDH, NM_001195426.1), 5′-TGAAGCAGGCGTCGGAGG G-3′ and 5′-CGAAGGTGGAAGAGTGGGTG-3′.

Rhesus monkey PCR primer pairs were purchased from Integrated DNA Technologies, Inc. (Coralville, IA, USA). Fidelity of the PCR reactions was determined by melting temperature analysis. All samples were run in triplicate for each gene. The relative gene expression was calculated using the ∆∆CT method normalized to the housekeeping gene GAPDH, whose expression remained constant in all experimental conditions.

### cDNA microarray analysis

RNA was isolated from rhesus monkey temporal neocortex at 24-months of dietary intervention using Trizol reagent (Invitrogen, Carlsbad, CA, USA) and further purified using RNeasy mini columns (Qiagen). RNA was processed, labeled and hybridized to Illumina HumanHT-12 V4.0 expression beadchip using standard Illumina protocols. Raw data were subjected to Z normalization; individual genes with Z ratio > 1.5 in both directions, *P* value < 0.05, and false discovery rate > 0.3 were considered significantly changed. The number of samples for each group was: SD =4, HFS =10, and HFS+R =10. Our expression data was tested for gene set enrichment using the parametric analysis of gene set enrichment (PAGE) method [76]. For each Z (pathway), a *P* value was also computed in JMP 6.0 to test for the significance of the Z score obtained. These tools are part of DIANE 6.0 and are available at http://www.grc.nia.nih.gov/branches/rrb/dna/diane_software.pdf.

### Protein extraction, gel electrophoresis and Western blotting

Frozen brain cortical tissues were homogenized in ice-cold lysis buffer [31], and clarified lysates were processed for SDS-polyacrylamide gel electrophoresis under reducing conditions with proteins transferred to nitrocellulose membranes. The membranes were routinely stained with the Ponceau S dye (Sigma-Aldrich, St-Louis, MO, USA) for the rapid and reversible detection of protein bands. Protein expression was detected using specific primary antibodies. Antibodies raised against Bcl2, cleaved caspase 3, FOXO3a, pVHL, acetyl-α-tubulin and phospho-p65Rel (Ser 536) were purchased from Cell Signaling Technology. (Danvers, MA, USA); NF-κB from Epitomics (Burlingame, CA, USA); eNOS from BD Biosciences (Franklin Lakes, NJ, USA); VEGF, ALDH2, SIRT2, β-actin, GAPDH and α-tubulin from Abcam; and HIF-1α from Cayman Chemicals (Ann Arbor, MI, USA). All antibodies were detected with horseradish peroxidase-conjugated secondary antibodies (Santa Cruz Biotechnology, Dallas, TX, USA) and visualized by enhanced chemiluminescence (GE Healthcare, Piscataway, NJ, USA). Quantitation of the protein bands was performed by volume densitometry using ImageJ software.

### Determination of dimeric and monomeric forms of eNOS

eNOS dimeric and monomeric species were separated, using low-temperature SDS–PAGE under reducing conditions followed by Western blot analysis, as described previously [**77**]. In brief, brain cortical samples were not heated and the temperature of the gel was maintained at 4°C during electrophoresis, which enabled the detection of the dimeric and monomeric forms of eNOS protein by immunoblotting.

### Hydrogen sulfide measurement

Hydrogen sulfide production was determined in protein lysates from cerebral cortex following the method described by Hine et al. [78]. In brief, brain tissues were homogenized in passive lysis buffer (Promega, Madison, WI, USA) followed by several rounds of flash freezing/thawing. After normalization for protein content with the bicinchoninic acid assay (Thermo Scientific Pierce, Rockford, IL, USA), 125 mg of lysates was added to a final reaction in 96-well format containing 10 mM cysteine and 1 mM pyridoxal 5′-phosphate. Pieces of lead acetate paper (15×10 cm) were made by soaking VWR^®^ grade 703 blotting paper (VWR International, Radnor, PA, USA) in 20 mM lead acetate (Sigma-Aldrich) and then vacuum drying, followed by their positioning over the 96-well dish and incubation for 18 h at 37°C until lead sulfide was detected but not saturated. Quantification was performed by volume densitometry using ImageJ software.

### Determination of IL-6 and RSV levels in CSF

IL-6 levels in CSF were determined using a specific monkey ELISA kit, according to the manufacturer's instructions (Invitrogen). The extraction of RSV from monkey CSF and its identification by mass spectrometric methods was undertaken as previously described [30].

### Statistical analysis

With regard to data analysis for western blotting, the nonparametric Kruskal-Wallis test was used to compare the three unmatched groups with different sample sizes (SD =4; HFS =10; HFS+R = 10). Rejection of the null hypothesis (p ≤ 0.05) was followed by Tukey post-hoc test to identify specific sample pairs that showed significant difference in mean ranks. P values less than 0.05 were considered statistically significant.

## SUPPLEMENTAL DATA



## Abbreviations

RSV: resveratrol
4-HNE: 4-hydroxynonenal
SD: standard diet
HFS: high-fat/high-sugar diet
HFS+R: HFS supplemented with resveratrol
eNOS: endothelial nitric oxide synthase
CSF: cerebral spinal fluid
VEGF: vascular endothelial growth factor
PCA: principal component analysis
PAGE: parametric analysis of gene-set enrichment
ROS: reactive oxygen species
GO terms: gene ontology terms
CNS: central nervous system
RT-PCR: real-time polymerase chain reaction
pVHL: von Hippel-Lindau protein
H2S: hydrogen sulfide
IL-6: interleukin 6
NF-κB: nuclear factor kappaB
COMMD: copper metabolism mURR1 domain containing
SIRT2: NAD^+^-dependent deacetylase 2
ALDH2: aldehyde dehydrogenase 2
TBI: trauma brain injury
