# Supplementary material for: Resveratrol supplementation confers neuroprotection in cortical brain tissue of nonhuman primates fed a high-fat/sucrose diet
Source: Aging (Albany NY). 2016 Apr 9;8(5):899–914. doi: 10.18632/aging.100942 (PMC4931843; doi:10.18632/aging.100942)
Supplement: Supplementary file 1 [file aging-08-0899-s001.pdf]

SUPPLEMENTAL DATA

Supplemental Table 1. List of shared GOTerms significantly enriched in both HFS_SD and HFS+R_HFS pairwise comparisons.

GoTerm	N genes	Z-score (HFS_SD)	Z-score (HFS+R_HFS)
GO0002062 CHONDROCYTE DIFFERENTIATION	6	4.2403	-2.2720
GO0030010 ESTABLISHMENT OF CELL POLARITY	7	3.3006	-2.4411
GO0030137 COPI COATED VESICLE	6	2.7585	-3.4888
GO0016272 PREFOLDIN COMPLEX	7	2.6731	4.2071
GO0050681 ANDROGEN RECEPTOR BINDING	20	-1.8098	2.9116
GO0004428 INOSITOL OR PHOSPHATIDYLINOSITOL KINASE	12	-1.9444	2.2001
GO0015288 PORIN ACTIVITY	30	-1.9502	2.8902
GO0030218 ERYTHROCYTE DIFFERENTIATION	18	-1.9730	1.7746
GO0007264 SMALL GTPASE MEDIATED SIGNAL TRANSDUCTION	210	-2.4826	4.6322
GO0004721 PHOSPHOPROTEIN PHOSPHATASE ACTIVITY	129	-2.5996	4.0727
GO0003723 RNA BINDING	536	-2.8103	4.7800
GO0006915 APOPTOSIS	417	-2.8134	2.1335
GO0018108 PEPTIDYL TYROSINE PHOSPHORYLATION	25	-2.8393	2.3433
GO0051028 MRNA TRANSPORT	64	-2.9635	3.6905
GO0007049 CELL CYCLE	462	-2.9651	4.1788
GO0006511 UBIQUITIN DEPENDENT PROTEIN CATABOLIC PROCESS	118	-2.9815	4.4421
GO0000070 MITOTIC SISTER CHROMATID SEGREGATION	12	-2.9990	2.0672
GO0006470 PROTEIN AMINO ACID DEPHOSPHORYLATION	127	-3.0079	3.9043
GO0042802 IDENTICAL PROTEIN BINDING	146	-3.0352	3.7537
GO0006357 REGULATION OF TRANSCRIPTION FROM RNA POL	194	-3.0606	3.9896
GO0016787 HYDROLASE ACTIVITY	1329	-3.1339	3.1600
GO0003700 TRANSCRIPTION FACTOR ACTIVITY	916	-3.2249	2.6877
GO0008285 NEGATIVE REGULATION OF CELL PROLIFERATION	181	-3.2625	4.0025
GO0004842 UBIQUITIN PROTEIN LIGASE ACTIVITY	128	-3.2880	3.1958
GO0016874 LIGASE ACTIVITY	269	-3.3764	3.8752
GO0005768 ENDOSOME	170	-3.6613	3.8803
GO0006810 TRANSPORT	1485	-3.6900	3.4847
GO0006974 RESPONSE TO DNA DAMAGE STIMULUS	170	-3.7341	2.6275
GO0005643 NUCLEAR PORE	67	-3.7788	4.1905
GO0008380 RNA SPLICING	188	-3.8151	3.0931
GO0006281 DNA REPAIR	196	-3.8201	3.6090
GO0006397 MRNA PROCESSING	229	-3.9536	4.1352
GO0005783 ENDOPLASMIC RETICULUM	658	-3.9581	5.0192
GO0006260 DNA REPLICATION	128	-4.0831	2.0360
GO0007399 NERVOUS SYSTEM DEVELOPMENT	289	-4.1914	2.3647
GO0005794 GOLGI APPARATUS	594	-4.2990	2.4954
GO0005622 INTRACELLULAR	1253	-4.4245	4.0702
GO0005681 SPLICEOSOME	135	-4.4959	3.2754
GO0015031 PROTEIN TRANSPORT	476	-4.7363	6.3000
GO0006512 UBIQUITIN CYCLE	384	-4.7990	5.8876
GO0005085 GUANYL NUCLEOTIDE EXCHANGE FACTOR ACTIVITY	112	-4.8455	3.3750
GO0046872 METAL ION BINDING	2047	-5.2149	4.2788
GO0008150 BIOLOGICAL PROCESS	1481	-5.3245	5.7086
GO0000166 NUCLEOTIDE BINDING	1586	-5.4279	2.4367
GO0005575 CELLULAR COMPONENT	1441	-5.5147	6.1531
GO0003674 MOLECULAR FUNCTION	1417	-5.7890	6.7542
GO0003677 DNA BINDING	1676	-6.0118	2.4020
GO0003676 NUCLEIC ACID BINDING	768	-6.0565	4.3375
GO0016020 MEMBRANE	4837	-6.3726	4.1302
GO0016021 INTEGRAL TO MEMBRANE	3894	-6.4015	2.3046
GO0008270 ZINC ION BINDING	1568	-6.6694	4.6477
GO0006350 TRANSCRIPTION	1380	-6.8999	4.6749
GO0006355 REGULATION OF TRANSCRIPTION DNA DEPENDENT	1670	-7.0525	4.4861
GO0005737 CYTOPLASM	3237	-7.5834	6.1063
GO0005515 PROTEIN BINDING	4416	-8.1867	6.5947
GO0005634 NUCLEUS	3662	-10.9629	7.7421

All GOTerms were statistically significant with Z-score >1.5 in either direction, p<0.05 and false discovery rate <0.3. See Materials and Methods for additional details. Red font: Up-regulated GOTerms; blue font: Down-regulated GOTerms; yellow cell: Lone GOTerm significantly increased by both pairwise comparisons.

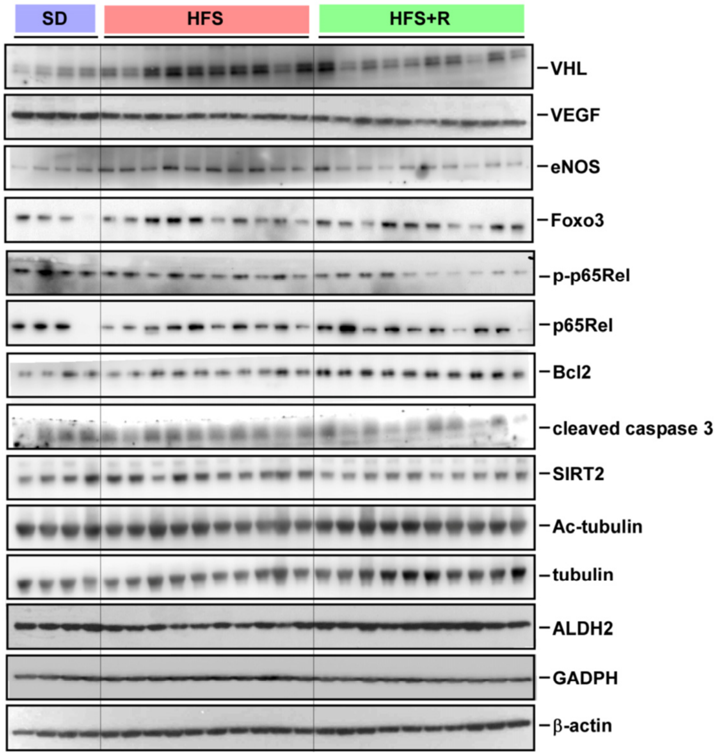

Supplemental Figure 1. Immunoblots used for quantitative analysis. Solubilized cerebral cortex extracts from SD-(n=4), HFS- (n=10) and HFS+R-fed animals (n=10) were resolved by SDS-PAGE under reducing conditions, electrotransferred onto nitrocellulose membranes and subjected to immunoblotting using the indicated primary antibodies. Probing membranes with GADPH and β-actin demonstrated equal loading in each lane. Bands of interest were quantitated by densitometry and normalized to Ponceau S staining of the membranes.
